# Supplementary material for: MetaRibo-Seq measures translation in microbiomes
Source: Nat Commun. 2020 Jun 29;11:3268. doi: 10.1038/s41467-020-17081-z (PMC7324362; doi:10.1038/s41467-020-17081-z)
Supplement: Supplementary file 10 — Supplementary Data 7 [file 41467_2020_17081_MOESM10_ESM.zip › File2/Confidence_High_Taxonomy/285923_out.krona.html]

Javascript must be enabled to view this page.

members
magnitude
magnitudeUnassigned
count
unassigned
taxon
rank

285923\_out

2

superkingdom
2
1

phylum
1224
1

1236
class
1

order
135619
1

family
28256
1

genus
2745
1

1

SRS1041145\_contig\_number\_33560
2498859
species


SRS098571\_contig\_number\_67853
1
